# Physical environment features that predict outdoor active play can be measured using Google Street View images

**DOI:** 10.1186/s12942-023-00346-3

**Published:** 2023-09-28

**Authors:** Randy Boyes, William Pickett, Ian Janssen, David Swanlund, Nadine Schuurman, Louise Masse, Christina Han, Mariana Brussoni

**Affiliations:** 1 Department of Public Health Sciences, Queen’s University, Kingston, ON K7L 3N6 Canada; 2 Presage Group, Inc, 3365 Harvester Road, Suite 206, Burlington, ON L7N 3N2 Canada; 3 Faculty of Applied Health Sciences, Brock University, 1812 Sir Isaac Brock Way, St. Catharines, ON L2S 3A1 Canada; 4 School of Kinesiology and Health, Queen’s University, Kingston, ON K7L 3N6 Canada; 5 Department of Geography, Simon Fraser University, RCB 6119/7134, Burnaby, BC V5A 1S6 Canada; 6 School of Population and Public Health, University of British Columbia, British Columbia Children’s Hospital, Room F508, 4480 Oak Street, Vancouver, BC V5H 3V4 Canada; 7 Department of Pediatrics, School of Population and Public Health, Human Early Learning Partnership, University of British Columbia, British Columbia Children’s Hospital, Room F511, 4480, Oak Street, Vancouver, BC V5H 3V4 Canada

**Keywords:** Child, Built environment, Social factors, Cities, Exercise, Play

## Abstract

**Background:**

Childrens’ outdoor active play is an important part of their development. Play behaviour can be predicted by a variety of physical and social environmental features. Some of these features are difficult to measure with traditional data sources.

**Methods:**

This study investigated the viability of a machine learning method using Google Street View images for measurement of these environmental features. Models to measure natural features, pedestrian traffic, vehicle traffic, bicycle traffic, traffic signals, and sidewalks were developed in one city and tested in another.

**Results:**

The models performed well for features that are time invariant, but poorly for features that change over time, especially when tested outside of the context where they were initially trained.

**Conclusion:**

This method provides a potential automated data source for the development of prediction models for a variety of physical and social environment features using publicly accessible street view images.

## Highlights

- This study describes an automated method for selecting StreetView images in a given area and extracting from them measures of the built and social environment.
- Measures obtained in this way are benchmarked against publicly available ground truth measures.
- The effectiveness of this method varies depending on the nature of the variable being measured.

## Background

Children’s outdoor active play is an important part of their healthy development, though not all children have equal access to it [1]. Outdoor active play provides children with an opportunity to be physically active and a chance to develop important social and emotional skills by interacting with their peers [2]. Children can only engage in outdoor play when they have a social and physical environment which is suitable for it. Key social factors include the availability of other children to play with, the trust of their parents to venture outside unsupervised, and their own feeling of safety with regards to the other people in the neighbourhood [3]. Furthermore, the physical environment needs to contain affordances, which are objects and spaces that can be used for play [1], and it needs to be safe from physical danger such as traffic and environmental hazards [4–6].

Measures of salient features used to describe outdoor play environments are typically only available in data sources held by local municipalities or not available at all. Traffic safety can be approximated using nationally available features such as road speed limit or easily calculated metrics such as street connectivity, but these features by themselves do not provide a full picture of street safety from the perspective of children and parents [7]. Pedestrian features such as sidewalks, bicycle paths, and crosswalks, traffic calming features such as speed humps and low speed zones, and traffic density all need to be considered. Children also play with and amidst other people, both adults and children, in urban street environments [8].

While the aforementioned environment features are difficult to measure across multiple jurisdictions with traditional data sources, there exists a large, easily accessible database of images from the perspective of the street for almost every location in most middle- and high-income countries: Google’s StreetView database [9]. Recent advances in deep learning architecture have made automated processing of these StreetView images possible [10]. This type of processing has already been used to identify “playability” directly from StreetView images, although interpretation such a model’s understanding of the concept of playability can be difficult [11]. This paper describes a method for extracting and processing active outdoor play environment features from large number of images for any area where StreetView data are available. While our current application for this process was to develop models for the measuring of environment features that predict of children’s outdoor active play behaviour, once the data are extracted and processed, they can be used to assist in the development of prediction models for any health outcome—such as asthma, obesity, mental health, or high blood pressure [12–14]—that is potentially influenced by the physical or social environment. Building an understanding of the relationship between our physical and social environment and these health outcomes can inform planning decisions to improve health outcomes across cities and neighbourhoods.

## Materials and methods

### Overview

This study is part of a larger project which aims to develop a national index of playability which can be used to predict the outdoor active play behaviour of children in neighbourhoods across Canada. The output of this process will be used across Canada as part of the calculation of playability. As this project’s outcome was measured at the level of the 6-digit postal code, all metrics from the models developed in this paper are also calculated at the level of the postal code and are made available at https://www.github.com/rdboyes/streetview. The method could be easily adapted to calculate metrics at different levels if required.

The process described below can be broken up into distinct stages. First, a set of points are chosen which cover the geographic area in question (i.e., the area covered by the postal code). Second, the available images from these points are queried from the Google StreetView image API. Third, a convolutional neural network is used to label the objects in the images. Fourth, the presence or absence of objects in the image is decided based on these labels using statistical models. Finally, data from all images obtained in the postal code area are aggregated to the level of the postal code for use as input in future prediction models.

### Populations and data sources

This study uses training data from Kingston, a small city of 150,000 people in Ontario, Canada and tests the resulting models using data from Vancouver a large city in British Colombia, Canada with a metropolitan area population of approximately 2.5 million. These cities were chosen due to proximity to study authors, their heterogeneous nature to ensure a fair out of sample test, and their availability of municipal data for use as ground truth when assessing the accuracy of the models. This ground truth data was obtained from open data portals and through direct requests to the cities.

The image data for comparison comes from Google’s StreetView image database via the static StreetView API using the R package googleway [15]. The static streetview API allows requests that include a latitude, longitude, and orientation (i.e. the direction the image is facing) and returns an image at the street level for the closest point available. Google StreetView images are not captured at a specific consistent distance apart; the distance between images depends on a few factors, including the equipment used, traffic at the time, and the location of the street. A typical pair of image capture locations would be approximately 25 m apart on a city street. Attempting to use all available images would not be feasible, both because of the extremely high number of images that would be required and because such a sampling strategy would result in many objects appearing in multiple images. Examination of a sampling of StreetView images suggested that the typical distance at which objects were still large enough to detect was approximately 75 to 100 m. Based on this, images were obtained at points every 150 m along every street in the study areas to obtain unique visual information on as many points as possible in each of the targeted postal code areas. For streets shorter than 150 m in length, a single point was queried at the midpoint of the street. At each point chosen by the sampling strategy, 4 images were obtained from the StreetView API corresponding to the cardinal directions. In cities, streets which do not have images are very uncommon, but if images were not available, the points were excluded from the analysis.

### Image processing

Our image processing strategy was designed to be able to classify each pixel in a StreetView image using categories which would be commonly seen in street-level images. These categories were taken from the labelling of the cityscapes dataset and include person, rider, sidewalk, road, vegetation, terrain, sky, pole, traffic light, traffic sign, car, truck, bus, motorcycle, and bicycle [16]. This gives information on the amount and location of each of these features visible in each of the images obtained from StreetView. Some of these categories were compared directly with available data, including traffic lights, traffic signs, trees, sidewalks, and people; others were collapsed, with bicycle and rider becoming “cyclists” and road, cars, trucks, and motorcycles being combined into “vehicles”; one was excluded because of its rarity in the data and lack of available comparison data (“bus”); and, finally, some were not examined as they had no direct comparison or were deemed unimportant for measurement of the playability environment (“terrain”, “sky”, and “pole”).

**Fig. 1:**
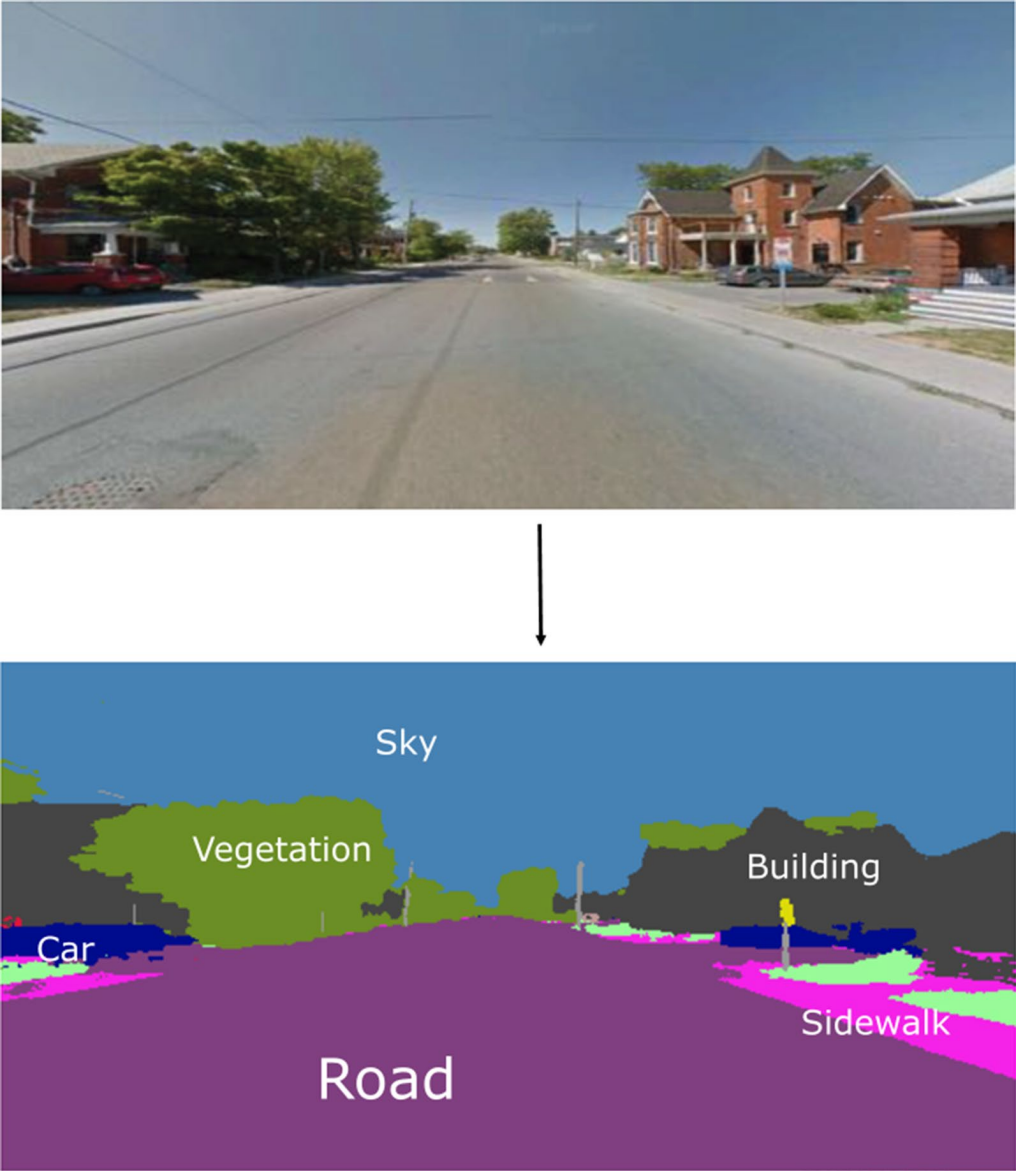
Example of pixel-level image segmentation of a representative Google StreetView image

To accomplish pixel-level segmentation, we used a deep neural network architecture originally proposed by DeepLab and implemented in tensorflow to analyze neighbourhood images [16]. The network consists of an encoder based on Xception [17] and a decoder which translates the features extracted by the encoder into pixel-level class predictions. The cityscapes datasets provide a small, finely annotated set consisting of 5,000 street-level images and a larger, coarsely annotated set consisting of 20,000 street-level images. Each of these datasets has pixel-level annotations for 19 classes of objects, including vegetation, vehicles, road, sidewalk, people, traffic signs, and traffic lights, anad others [18]. Using these datasets, neural networks previously optimized for detection of generic objects in the imagenet or other large datasets can be retrained to categorize common objects found on the street. The resulting model retains the lower-level layers which allow the recognition of features such as edges, colours, and textures, but interpret these features differently due to retraining of the later layers and the decoder. Our analysis uses a model checkpoint set by DeepLab. For the complete code used to implement all models and algorithms described in this paper, please refer to the github repository, found at github.com/rdboyes/streetview/. An example StreetView image segmentation is shown in Fig. 1.

### Units of analysis

For each feature we measured, we attempted to quantify the accuracy of the method as it would be used in the assessment of a neighbourhood. Where data were available and appropriate, we calculated a 500-m Manhattan-style buffer around each postal code centroid in the study area, and compared the assessment of each neighbourhood feature from image and from the standard data source. For measures of traffic density that were measured through a single point, buffers were sized in the training data based on the highest correlation with the ground truth data and tested in the test data for accuracy. In urban areas in Canada, six-digit postal code regions can be very small. The use of these buffers standardizes the amount of space covered to more accurately represent the walkable area in the immediate vicinity of a home in those postal codes.

### Geographic measurement

#### Trees

Vancouver and Kingston open data [19, 20] both provide the location of every tree visible from the street in the study area. The target measure for both cities was defined as the number of trees inside of each postal code manhattan buffer, which was predicted using the “vegetation” measure from the StreetView images in the same area.

#### Sidewalks

Kingston’s open data provides the locations of all town-maintained sidewalks maintained by the city. Each road segment in Kingston was classified using these data as either having a sidewalk or not. If sidewalks were detected in images on a road segment, then the road segment was considered to have a sidewalk according to the StreetView measure. Classification sensitivity and specificity were calculated for the StreetView measure using the municipal data as ground truth. For each postal code area, the target metric was the meters of road length with sidewalks, which was predicted based on aggregating the predictions at the segment level. Equivalent ground truth data were not available in Vancouver. This outcome was predicted using the “sidewalks” measure from the StreetView images.

#### Density of pedestrians, cyclists, and vehicles

Data for traffic counts through intersections were obtained via direct request from the city of Kingston and from the Vancouver Open Data portal. Traffic counts for Vancouver were available in some cases for multiple years, and in these cases, the most recent year’s data was used. Kingston’s measures were an annualized expected traffic count for pedestrians, cyclists, and vehicle traffic. Vancouver’s reported numbers were the total count of traffic first and last 2-h block of the day, representing rush-hour traffic. While these counts of traffic are measured at the level of an intersection, they require StreetView data from a larger area to measure correctly. Forty different buffer sizes were tested in Kingston, ranging from 50 to 2000 m around each of the 576 intersections for which data was available, were tested to determine the range required to obtain the best measure of traffic through intersections available from StreetView data; optimal buffer sizes were selected considering the tradeoff between size and model accuracy, and these same buffer sizes and models were tested in Vancouver. These outcomes were predicted using the “road”, “car”, “truck”, and “person” measures from the StreetView images.

#### Traffic lights and traffic signs

Ground truth traffic light and traffic sign data were obtained from the city of Kingston directly and from the Vancouver Open Data portal. The models developed with the Kingston data using the “pole”, “traffic sign”, and “traffic light” pixels in the StreetView images, and were then tested in the Vancouver data. The model in both Kingston and Vancouver targeted the number of traffic signs and traffic lights per postal code area.

### Statistical analysis

This study uses three different model architectures—a support vector machine, an XGBoost model, and a linear model—to test the viability of the StreetView point measure in assessing measures of the built environment for use in prediction models. The models use standardized inputs and outputs to increase the likelihood that the models will be transferable to different cities and contexts. Each model is evaluated using three metrics: root mean squared error (RMSE), R^2^, and mean absolute error (MAE).

Error statistics in both the training and test sets are provided. In the training set, error statistics are calculated using fivefold cross validation, while test set error statistics are simply calculated using the full test set. Data analysis was conducted in R version 4.2.1 using the tidyverse suite of packages for data processing and cleaning and the simple features package for handling geographic data [21–23]. The caret package was used for model fitting and evaluation [24]. Python was used for the neural network implementation, with the reticulate R package being used to communicate between the two languages when required [25].

## Results

The overall accuracy of the method using the best of the three models is summarized in Table 1. Model performance was highest in prediction of vegetation and sidewalks, moderate in traffic signs and lights, and lowest (especially out of sample) for pedestrians, bicycles, and vehicle traffic.

**Table 1:** Error statistics for the seven outcomes in each of the two cities (best model)

Outcome	Target	Train (Kingston)			Test (Vancouver)		
		RMSE	R^2^	MAE	RMSE	R^2^	MAE
Vegetation	Standardized Number of Trees (Postal Code (PC))	0.63	0.60	0.38	0.53	0.74	0.40
Sidewalks	Sidewalk Length (PC)	824	0.96	473	–	–	–
Pedestrians	Standardized Count (Intersection)	0.81	0.28	0.29	1.08	0.03	0.67
Bicycles	Standardized Count (Intersection)	0.83	0.27	0.44	1.77	0.00	1.08
Vehicles	Standardized Count (Intersection)	0.91	0.21	0.28	2.02	0.16	1.48
Traffic Lights	Standardized Count (PC)	0.51	0.74	0.29	1.29	0.27	0.97
Traffic Signs	Standardized Count (PC)	0.50	0.75	0.33	–	–	–

### Training data (Kingston, ON)

**Fig. 2:**
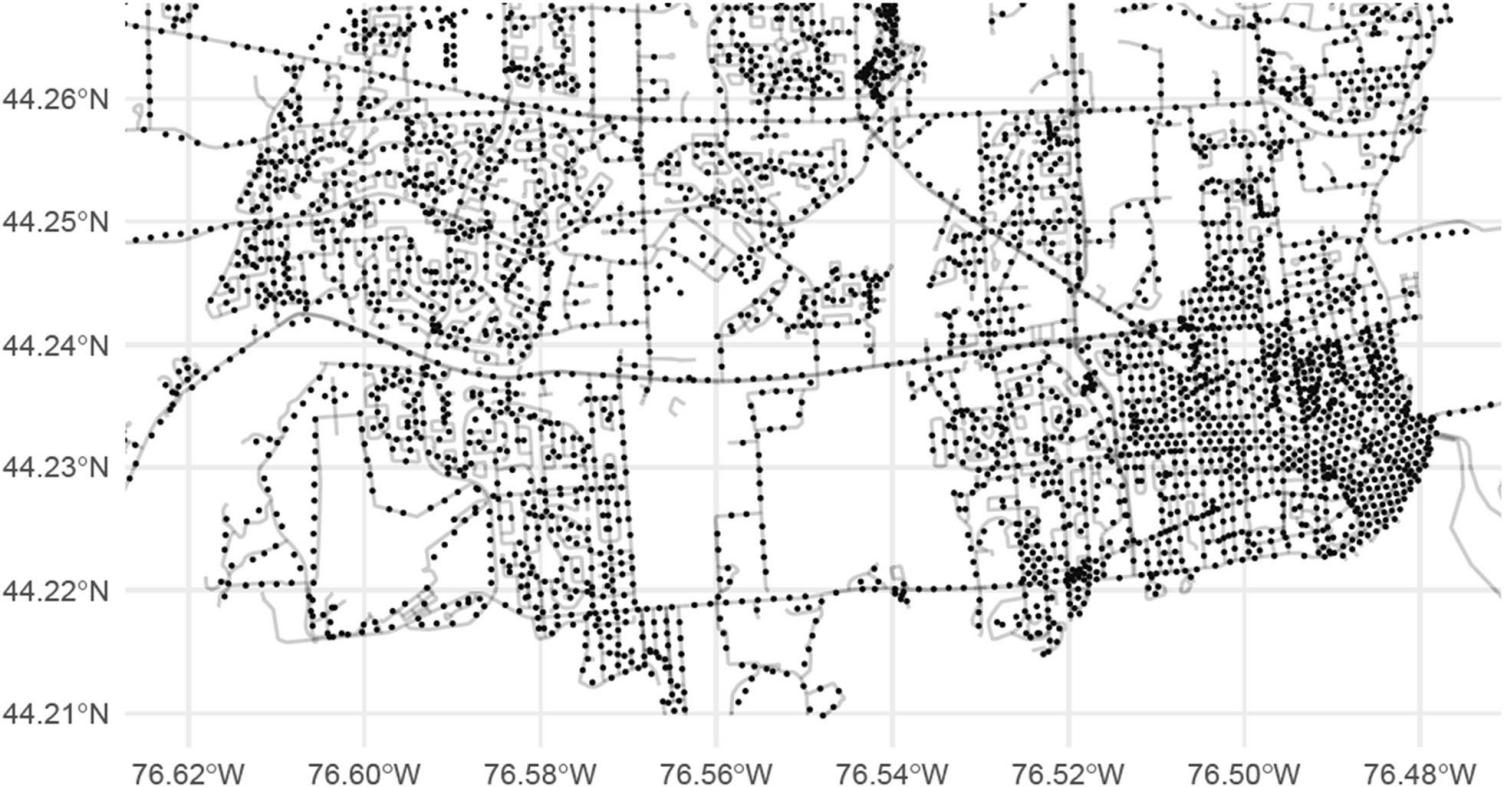
Points in Kingston, ON which were measured using Google's StreetView API

Kingston’s sample consisted of 4,270 points. The section of Kingston from which points were obtained ranged from longitude 76.625W to 76.478W and from latitude 44.21N to 44.265N, which was chosen to capture the section of the city with high population density and a high concentration of residential streets (Fig. 2).

#### Vegetation, sidewalks, and traffic lights and signs

**Fig. 3:**
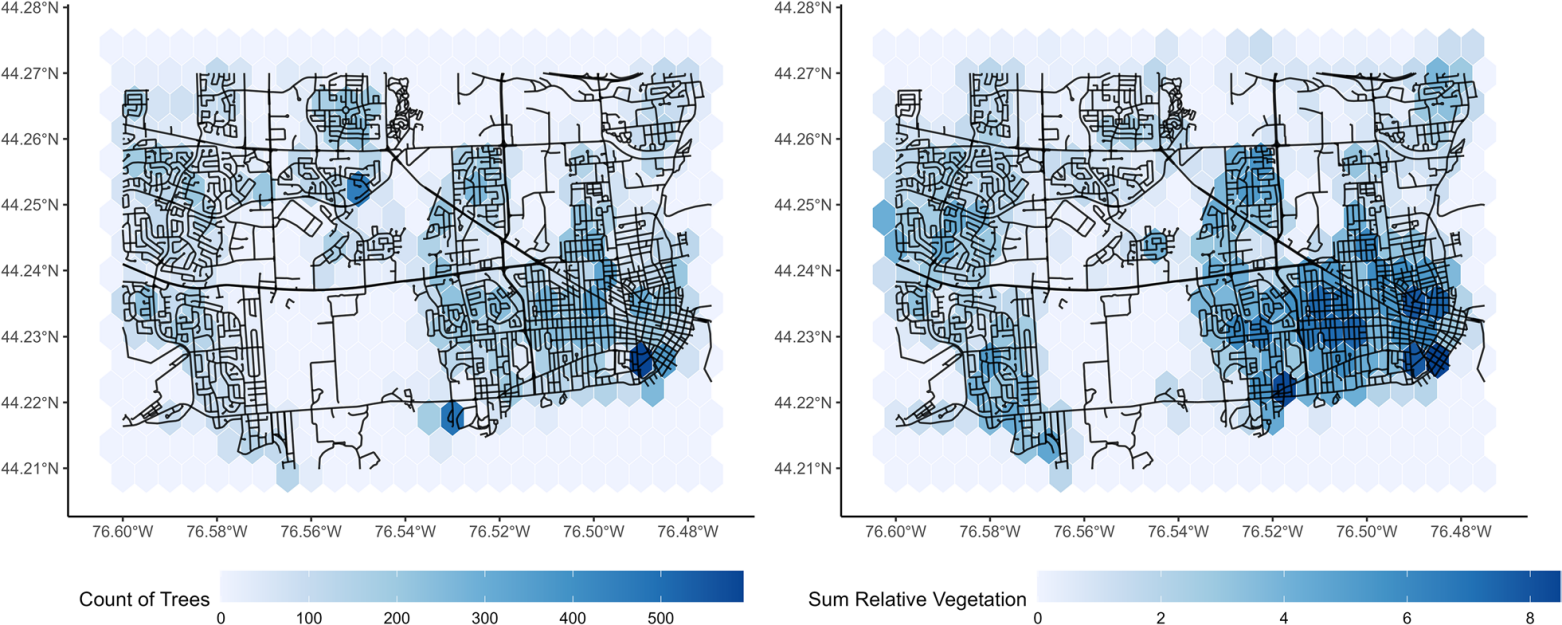
Comparison between vegetation pixel counts and true count of trees in the study area

The vegetation measure corresponds well to the number of trees present in each postal code zone in Kingston, with a RMSE of 0.63, an R^2^ of 0.60, and a MAE of 0.38. There is little geographic variation in the accuracy of the prediction. Linear and XGBoost-based models show similar performance. A comparison between the StreetView measure and the ground truth data is displayed in Fig. 3.

**Fig. 4:**
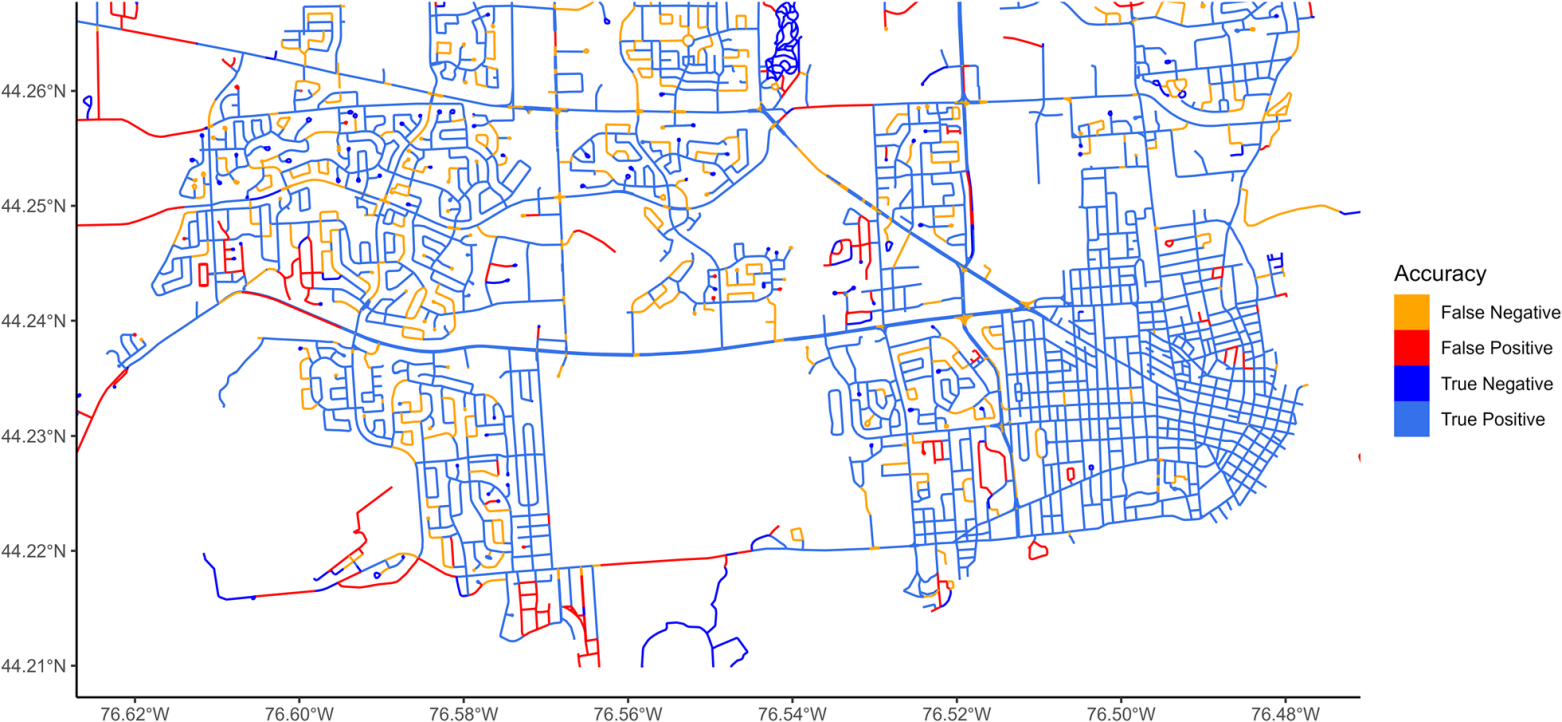
Classification accuracy for sidewalks in the study area

Per metre of road, sidewalks were measured with sensitivity of 0.90 and specificity of 0.65. Further accuracy metrics and a confusion matrix are presented in Table 2. Graphical examination of the sidewalk’s classification accuracy shows marked differences in accuracy across geographical areas (Fig. 4). On residential roads, overall accuracy is good, with many segments being correctly classified as true positives. Within, some segments are missed, as the sidewalk is obscured from view, most commonly by parked vehicles. Most false positives are on rural roads with high speed limits or highways, where the paved shoulder is mistaken for sidewalk.

**Table 2:** Error statistics for each model type for outcomes with constant buffer sizes

Target	Method	RMSE	R^2^	MAE
Vegetation	Linear model	0.65	0.58	0.39
	XGBoost	0.65	0.58	0.39
	SVM	0.63	0.60	0.38
Sidewalks	Linear model	814	0.95	474
	XGBoost	1863	0.80	529
	SVM	824	0.96	473
Traffic lights	Linear model	0.61	0.63	0.38
	XGBoost	0.51	0.74	0.29
	SVM	0.66	0.62	0.37
Signed intersections	Linear model	0.56	0.68	0.40
	XGBoost	0.50	0.75	0.33
	SVM	0.57	0.68	0.40

**Fig. 5:**
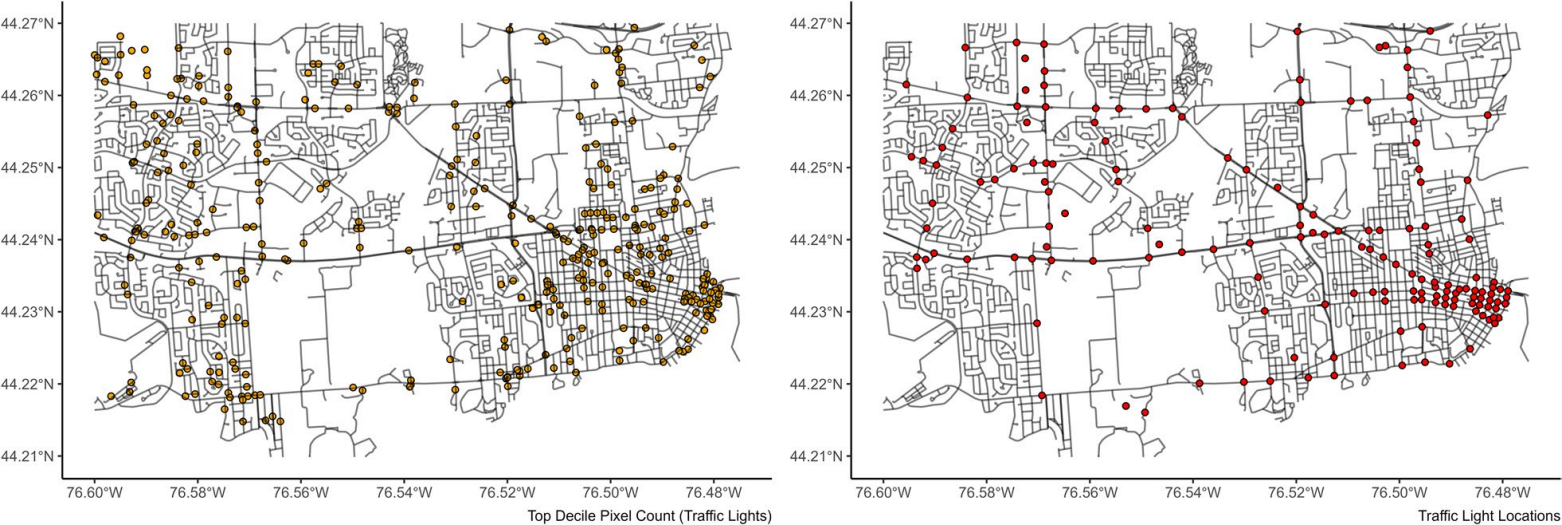
Top decile of pixel count for traffic lights compared with true locations of traffic lights in the study area

**Fig. 6:**
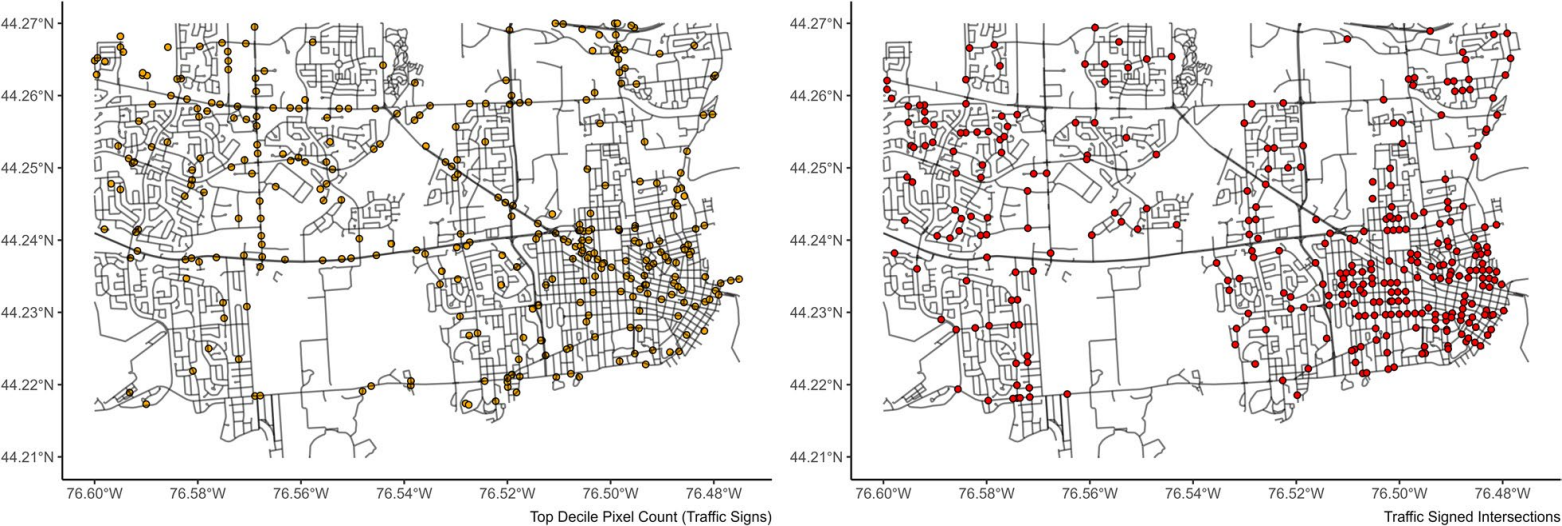
Top decile of pixel counts for traffic signs compared to locations of traffic signs at intersections in the study area

The standardized number of intersections with traffic signs and traffic lights correlated well with the model’s predictions in the training data (Table 2). Error rates were comparable across model types, with XGBoost models performing best for prediction for both types. Visual comparisons of the locations of highest pixel counts of traffic lights and signs compared to the locations of intersections with traffic lights and traffic signs are presented in Figs. 5 and 6.

#### Vehicles, bicycles, and pedestrians

**Fig. 7:**
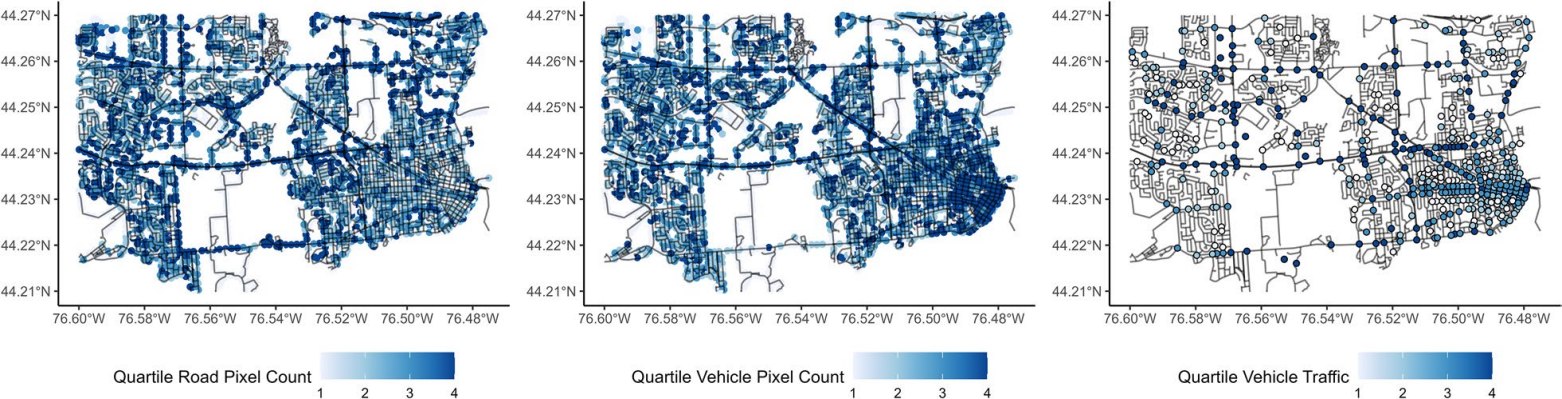
Pixel counts of car and road compared to vehicle traffic count in the study area

Training data from Kingston were annualized rates of traffic through any given intersection for 502 intersections within the study area of interest. The size of buffer used to capture image data for vehicles, bicycles, and pedestrians was allowed to vary. The vehicle measure is most highly correlated with true traffic counts when measured in a small area around the intersection in question; a 500-m buffer with an XGBoost model was found to be optimal in our sample. Contrary to expectations, when examining the variable importance in the model and the raw correlations, the observed number of vehicles in images is surprisingly inversely related to the traffic in an area (Fig. 7). We expect that this relationship is observed due to the high prevalence of vehicles parked on the side of the road in images, and lower traffic in areas where this is the case. However, traffic can still be measured using the inverse of the amount of visible road present in images rather than vehicles, as more visible road necessarily means fewer driving vehicles.

**Fig. 8:**
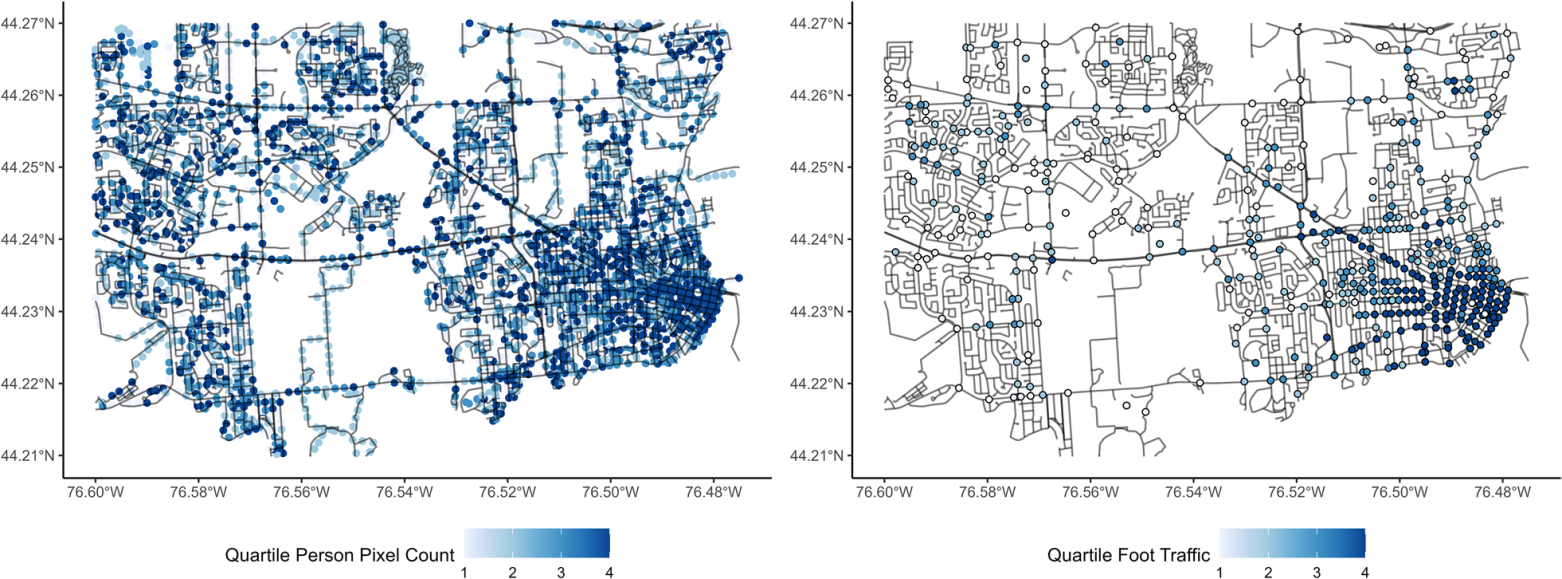
Quartile of person pixel count compared to quartile of pedestrian traffic in the study area

**Fig. 9:**
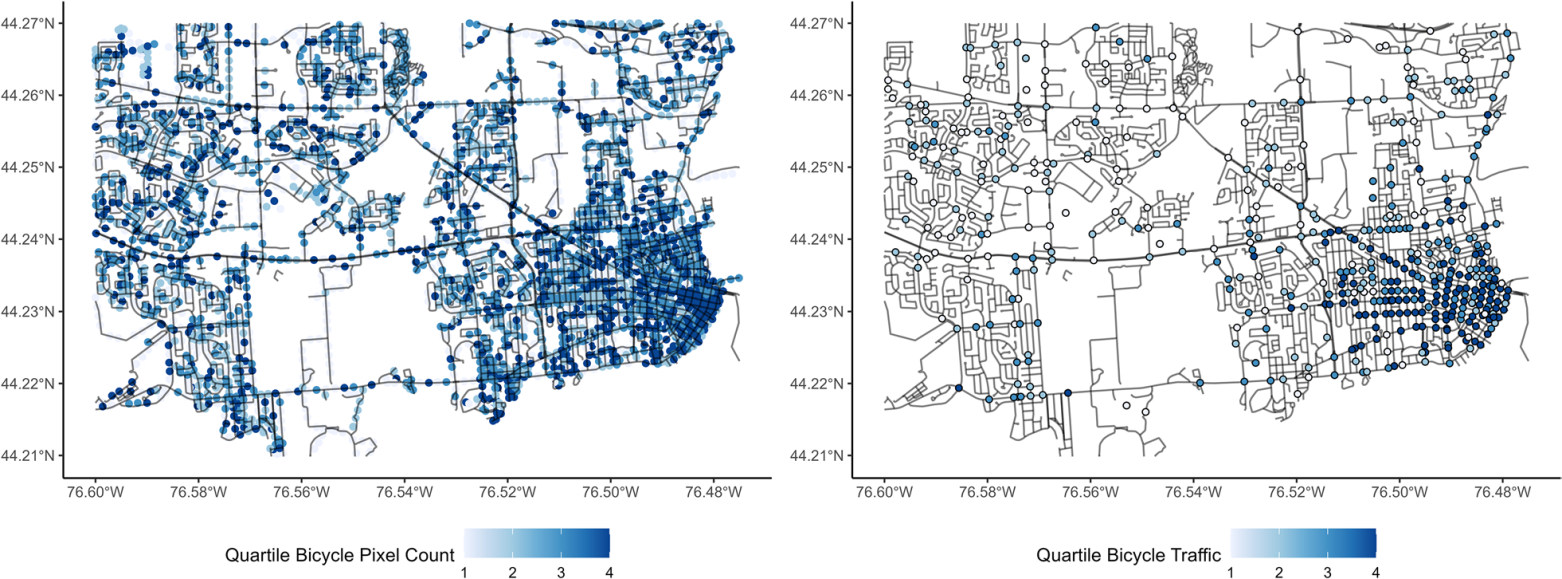
Quartile of bicycle pixel count compared to quartile of bicycle intersection traffic in the study area

Bicycles and pedestrians are less common in images than vehicles, and as such, increased buffer sizes resulted in better measurements of pedestrian and cyclist traffic. Buffer sizes of 1000 m were found to be ideal in the training data for both pedestrians and cyclists, with XGBoost performing better for bicycles and SVM performing better for pedestrians. The accuracy metrics for each of these variables are presented in Table 3. Pixel count data are compared with intersection traffic data in Figs. 8 and 9.

**Table 3:** Error statistics for each model type for outcomes with varying buffer sizes

Target	Best method	Best buffer	RMSE	R^2^	MAE
Bicycles	XGBoost	1000 m	0.83	0.27	0.44
Vehicles	XGBoost	500 m	0.91	0.21	0.69
Pedestrians	SVM	1000 m	0.81	0.28	0.29

### Test data (Vancouver, BC)

**Fig. 10:**
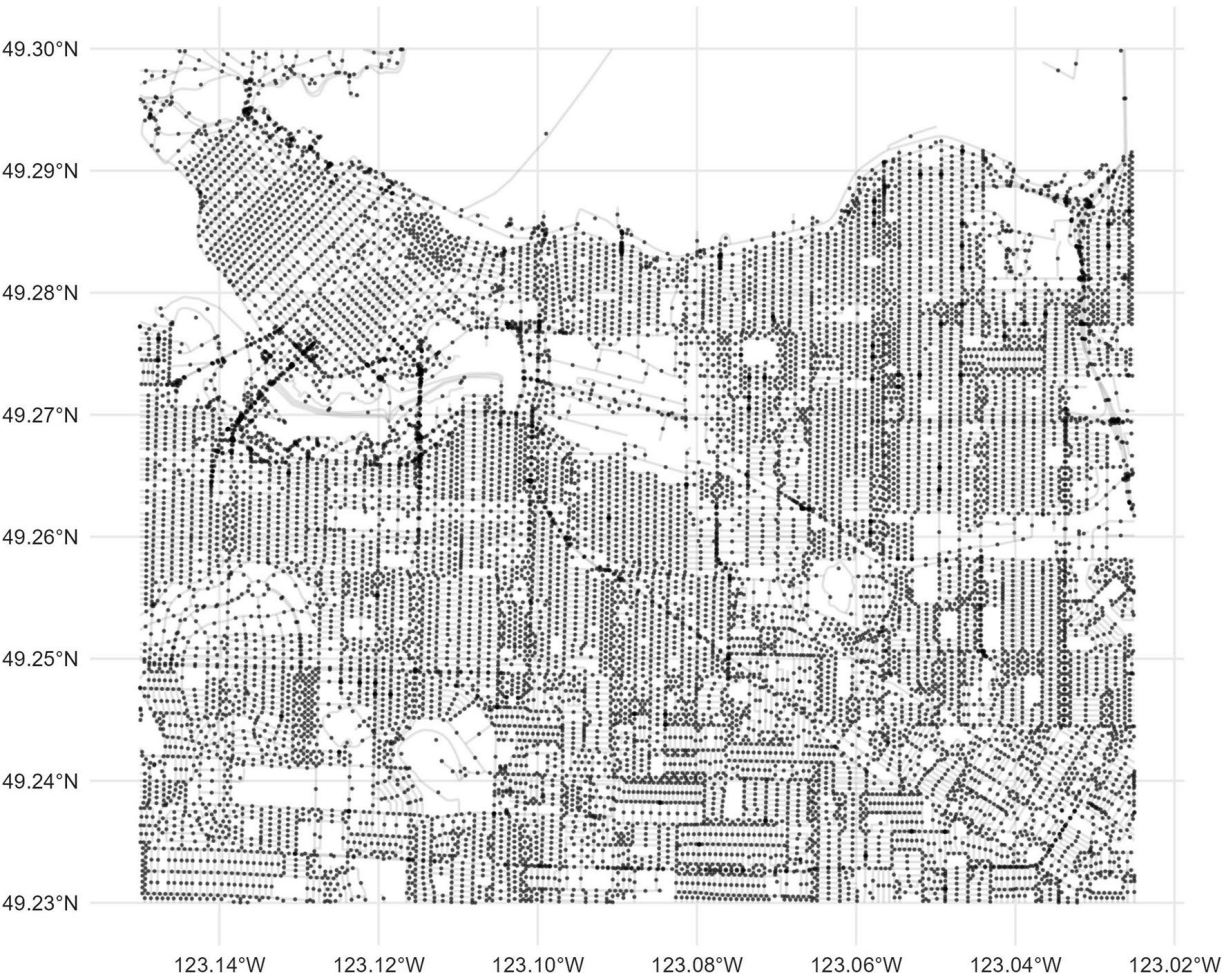
Points in Vancouver where StreetView image data were obtained from the StreetView API

The testing data for the models trained in the Kingston data come from a section of Vancouver between 123.15W and 123.02W, and 49.23N and 49.30N. A total of 14,858 points were queried in this area (Fig. 10). Ground truth data for the presence or absence of sidewalks and signed intersections was not available for Vancouver, but the remaining outcomes were available.

Predictions of the standardized number of trees inside Vancouver postal code areas were made using the StreetView points queried in Vancouver and the predictions model trained on Kingston data. The best performer on the training set, which was the SVM model, performed well on the new data, with a RMSE on the standardized scale of 0.48, an R^2^ value of 0.78, and a MAE of 0.37. The model was well calibrated, with the difference between the average predicted score and average observed score equal to 0.026. Model accuracy for traffic lights was lower in Vancouver than in the training set, with RMSE increasing to 1.57, MAE increasing to 1.05, and R^2^ decreasing to 0.23. Model accuracy decreased significantly for the measures of bicycle, car, and pedestrian traffic between the Kingston data and the Vancouver data, with nearly all predictive utility being lost in the test set. Full accuracy metrics are presented in Table 4.

**Table 4:** Test error statistics for the best performing model in the training data

Target	Best method	Best buffer	RMSE	R^2^	MAE
Vegetation	SVM	-	0.48	0.78	0.37
Traffic lights	XGBoost	-	1.57	0.23	1.05
Bicycles	XGBoost	1000 m	1.77	0.00	1.08
Vehicles	XGBoost	500 m	2.02	0.16	1.48
Pedestrians	SVM	1000 m	1.08	0.03	0.67

## Discussion

This study demonstrates the potential for the augmentation of existing methods for measurement of features of the physical environment that predict outdoor play using Street View image data. This method could be particularly useful when other sources of data are not available for the desired environmental measure. These methods are particularly helpful for features with less variability over time, such as the presence or absence of sidewalks, the amount of trees near the road, and traffic lights or signs. Images can still provide measures of time varying features, but they should be used with caution as they may be less representative of the true values for a neighbourhood.

The methods and data used in this study have potential for use in prediction models and indices of any health outcome that is influenced by the physical environment, which could allow these indices to expand their geographic areas more easily or have access to predictor variables that they would otherwise not have available. Indices for measuring the appropriateness of space for running such as the runnability index [26] could incorporate information on the presence or absence of sidewalks. A measure of the presence of trees could inform a predictive model for mental health outcomes [27]. Air quality measures, which have numerous health impacts, could incorporate traffic data from images [28]. This data source also provides the ability to test, supplement, and/or automate data collection for established indices of walkability or cyclability to determine the degree to which people are the degree to which people are actualizing the affordances in the environment for physical activity [29–31]. These measures are imperfect but provide a meaningful improvement over not having any measure at all.

This study provides an alternative measure for some important features of children’s physical environment. By using Google’s API as the primary data source, this method provides relatively current data with coverage of as much as 99% of the world. However, the nature of this data means that the measures taken of a neighbourhood are taken at a single time point, usually on a summer afternoon. These data cannot provide information about seasonal variability or variability throughout the day. The rate at which StreetView images are updated is not uniform across countries and is slower in rural areas than in urban centres. This could create bias if this method was used to compare across countries or to compare urban and rural areas, as some images would be more out of date than others. Getting the most accurate measurements possible is computationally expensive. The small sections we have examined here took approximately 24 h per 10,000 points using a Windows PC with a 8^th^ generation intel i5 at 2.8 GHz and a NVIDIA 1080 GPU to query the StreetView API and run the neural networks for segmentation. With a small city such as Kingston, this may not be an issue, as only 5,000 points were needed, but the part of Vancouver which was examined consisted of 15,000 points; far more would be needed for the whole city or if one wished to compare multiple cities. In addition, API queries at this scale can become expensive. Google’s StreetView API currently charges 7 USD per 1,000 queries.

Reductions in point density using grid-based approaches were attempted and the effect on accuracy was observed. These tests restricted the number of points sampled by covering the bounding box of the area in question with a hexagonal grid of varying cell size from 50 m across to 500 m across, then taking at most one point from each cell in the grid. This method allowed reductions in sampled points ranging from minor (~ 10%) to significant (~ 75%). Even at the lowest levels of point reduction, this method resulted in large decreases in accuracy of measurement which far outweighed the computational savings. We therefore recommend that for this method to be used, a density of images at least as high as the density used in the baseline case is likely necessary.

While this study demonstrates the correlation between measures derived from StreetView images and more traditional measures, it remains to be demonstrated if these features are useful for the prediction of children’s outdoor active play. Ideally, these features capture a different aspect of the neighbourhood features in question and improve the accuracy of prediction models built using both the traditional data sources and these image-derived data. Future work can implement prediction models for additional outcomes based on these technique**s** and evaluate their usefulness for prediction of diverse questions.

## Conclusions

The use of machine learning methods to make non-traditional data sources available for epidemiological research remains relatively unexplored. This study demonstrates the potential usefulness of automatically processed images as supplemental data for geographic prediction models for public health outcomes.

## Data Availability

This study relies on data which are available publicly at the open data portals for Kingston (https://opendatakingston.cityofkingston.ca/pages/welcome/) and Vancouver (https://opendata.vancouver.ca/pages/home/) as well as data which are available though Google’s StreetView API (https://developers.google.com/maps/documentation/streetview/overview). The authors have provided the code used to process these data (github.com/rdboyes/streetview), but have not rehosted the data required.
